# Large tumor size is a poor prognostic factor of gastric cancer with signet ring cell: Results from the surveillance, epidemiology, and end results database

**DOI:** 10.1097/MD.0000000000017367

**Published:** 2019-10-04

**Authors:** Liyuan Zhou, Weihua Li, Shaoxin Cai, Changshun Yang, Yi Liu, Zhizun Lin

**Affiliations:** a Department of Surgical Oncology, Fujian Provincial Hospital; b Shengli Clinical Medical College of Fujian Medical University, Fuzhou, China.

**Keywords:** gastric cancer, lymph node metastasis, signet ring cell, tumor node metastasis classification

## Abstract

There has been a steady increase in the incidence of signet ring cell (SRC) carcinoma, a distinct histological type with cells containing abundant intracytoplasmic mucin. We aimed to analyze the clinicopathological characteristics and prognostic value of patients with SRC gastric cancer (GC) who underwent gastrectomy.

Clinical data of 10,312 GC patients who underwent D2 radical gastrectomy were obtained from the Surveillance, Epidemiology, and End Results database and were retrospectively analyzed. X-tile plots were constructed to illustrate the optimal cut-off points using the minimum *P*-value from the log-rank Chi-squared test. The Kaplan–Meier method was used for the analysis of the overall cumulative probability of survival. Their differences were evaluated using the log-rank test. The Cox multiple factors analysis was performed using the logistic regression method.

In total, 946 (9.17%) SRC GC patients with pT1a-4bN0-3bM0 stage cancer were recruited. The optimal cut-off point for size was 49 mm. The 3-year overall survival (OS) rates of the SRC GC, large-size, and small-size groups were 35.89%, 30.63%, and 44.96%, respectively (*P* < .05). Cox multivariate analysis showed that tumor size (odds ratio [OR] = 2.032), T3 category (OR = 1.324), T4a category (OR = 1.945), and T4b category (OR = 2.163) were independent hazard prognostic factors.

SRC GC has a distinct biological behavior, presents as a large-sized tumor (≥49 mm), and is associated with worse outcomes. SRC GC patients have 2.032 times risk of mortality. SRC patients with larger tumors are at higher risk for infiltrative growth, lymph node metastasis, and distant metastasis.

## 1 Introduction

Gastric cancer (GC) can be classified histologically into various types.^[1]^ Although the incidence of GC has decreased, the incidence of signet ring cell (SRC) carcinoma remained high.^[2]^ SRC is a distinct histological type with cells containing abundant intracytoplasmic mucin,^[3]^ and its characteristic ring appearance is due to its mucin-rich cytoplasm and crescent-shaped nucleus. According to the Japanese Classification System,^[4]^ SRCs of the stomach are classified as undifferentiated. However, according to Lauren classification, SRCs of the stomach are classified as diffuse.^[5]^

In theory, patients with positive lymph nodes (LNs) have a worse outcome. Several GC patients with node-positive disease die as a result of postoperative recurrence and metastasis.^[6–8]^ However, only a few studies have reported on the clinicopathological features and prognosis of patients who developed SRC of the stomach with positive LNs. Most of them had the following limitations: the study used a small sample size, multivariate analysis was not conducted, and the study was restricted to a specific patient group.

To address the abovementioned concern, we aimed to investigate the clinicopathological characteristics and prognostic value of SRC in patients with gastric cancer using the clinical data of gastric cancer patients from the Surveillance, Epidemiology, and End Results (SEER) database.

## 2 Methods

### 2.1 Ethics

Ethics committee of Fujian Provincial Hospital reviewed and approved this study. Data of this study were searched from SEER database. This study followed SEER Research Data Agreement and we have got approval from SEER^∗^Stat (approval number: 15081-Nov2017) for accessing and using data in SEER database.

### 2.2 Patients

A total of 10,312 GC patients were recruited from the SEER database between 2004 and 2011. Among them, 946 patients with SRC GC who underwent resection were identified. A detailed description of the associations between lymph node metastasis status and clinicopathological characteristics are presented in Table 1.

**Table 1:**
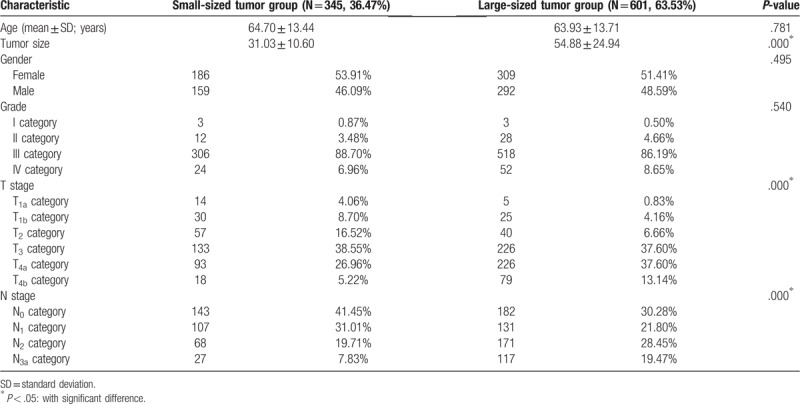
Demographics and clinicopathological characteristics of patients with gastric signet ring cell carcinoma.

Patients with the following characteristics were included: presence of GC, SRC confirmed by histopathology, baseline health status suitable for surgery, and with no prior history of any type of adjunctive therapy. In contrast, patients with the following characteristics were excluded: those with a history of cancer or with another type of cancer, those with a history of or concomitant gastrectomy for benign disease, patients with a history of receiving chemotherapy or radiotherapy, those with esophageal involvement, distant metastatic disease, multiple primary malignancies, remnant GC, and those who died within 30 days after surgery.

### 2.3 Clinicopathological characteristics

The clinicopathological findings, including depth of tumor invasion and LN metastases, were utilized to stage tumors according to the National Comprehensive Cancer Network guidelines, 8th edition. LNs were dissected and described according to the Japanese Classification of Gastric Carcinoma, which was also used to classify the location, histological type, and lymphatic invasion of tumors.

### 2.4 Statistical analysis

The associations between tumor size and clinicopathological features were analyzed using a Chi-square test. Risk factors for survival outcomes were identified by Kaplan–Meier analysis and Cox regression models. Only those variables that were considered significant in the univariate analysis were included in the multivariate model. The primary endpoint of this study was cause-specific survival. Deaths attributed to gastric cancer were treated as events, while other reasons associated with death or survival were defined as censored events. All analyses were performed using the R survival package (Version 3.2.1, Comprehensive R Archive Network, The R Foundation for Statistical Computing, Wirtschaftsuniversität Wien Welthandelsplatz 1 1020 Vienna, Austria) and SPSS (Version 22.0, IBM Corporation, 1 New Orchard Road. Armonk, NY). Prism 5 for Windows (Version 5.01, GraphPad Software, Northside Dr. Suite 560, San Diego, CA) was used to draft the Kaplan–Meier curve. A *P*-value of <.05 was considered as significant.

## 3 Results

### 3.1 Identification of size cut-off points

Using a minimum *P*-value from the log-rank Chi-square test, the X-tile plot illustrated that the optimal cut-off point for tumor size in SRC GC patients categorized as pT1a-4bN0-3bM0 was 49 mm. Based on this, the patients were divided into 2 groups, the small-sized tumor group and the large-sized tumor group, with the strongest discriminatory capacity.

### 3.2 Clinicopathological characteristics

A total of 946 SRC GC patients categorized as pT1a-4bN0-3bM0, which accounted for 9.17% (946/10,312) of all GC patients, were eligible for final analysis. Approximately 462 (48.84%) deaths were reported in a median follow-up of 27.69 months (range: 1–47 months). A detailed description of the associations between tumor size and clinicopathological characteristics are presented in Table 1. Compared with small-sized SRC, large-sized SRC frequently occurred in patients within the T4 category (50.74% vs 32.18%) (*P* < .05).

### 3.3 Survival analysis

**Figure 1:**
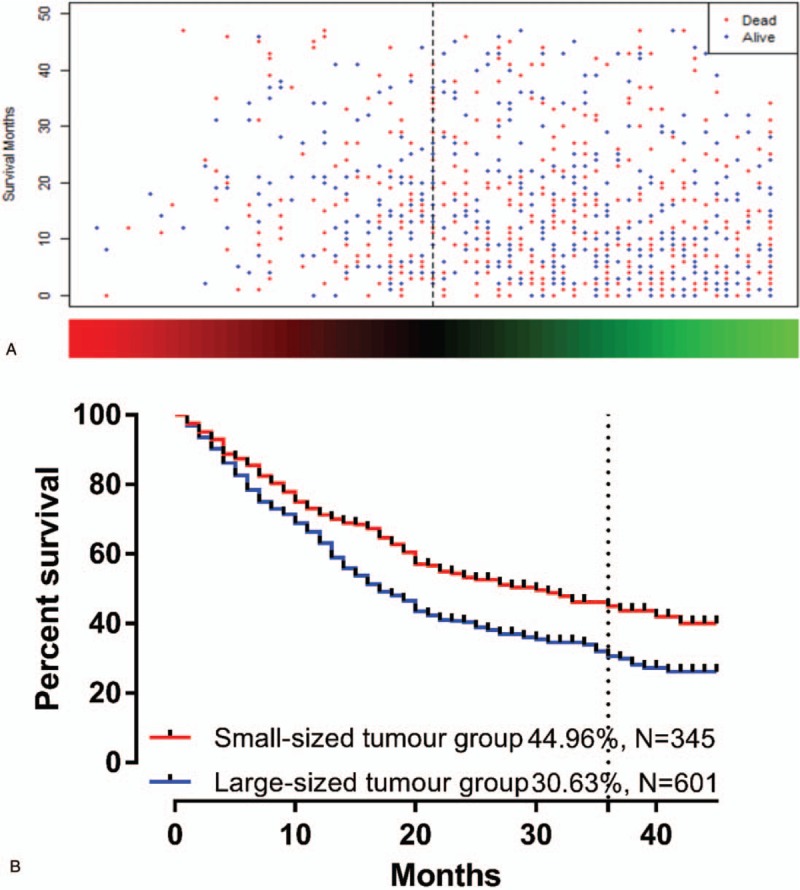
Overall survival rates of SRC gastric cancer patients. The distribution of survival status was created using the linear combination of the estimated regression coefficients derived from tumor size (A). The survival curve of the 2 groups is shown in B, which indicated that those in the large-sized tumor group had poorer prognoses than those in the small-sized tumor group (44.96% vs 30.63%, *P* < .05). *P*-values were calculated using the log-rank test. SRC = signet ring cell.

The survival distribution was built using the linear combination of the estimated regression coefficients derived from tumor size (Fig. 1A). The 3-year overall survival (OS) rate of SRC GC cases was 35.89%. The survival curve of the 2 groups is shown in Fig. 1B, which demonstrated that the large-sized tumor group had poorer prognosis than the small-sized tumor group (44.96% vs 30.63%, *P* < .05).

### 3.4 Cox multivariate analysis

**Figure 2:**
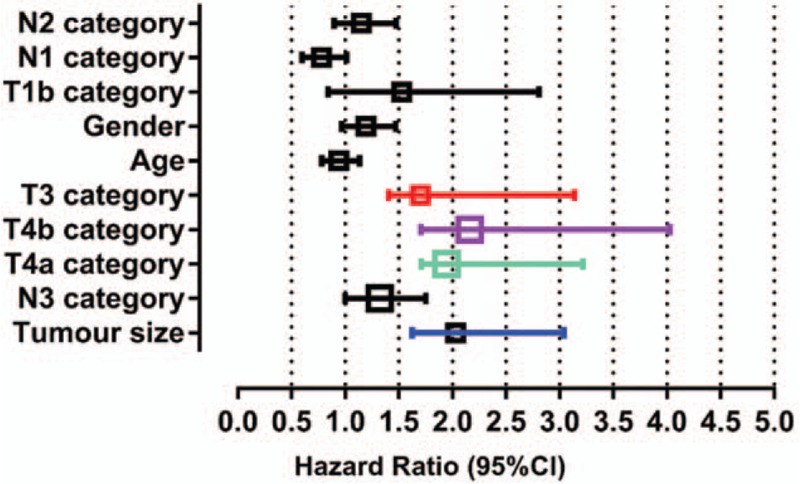
Independent prognostic factors were determined by multiple Cox regression analysis. Multivariate analysis showed that tumor size (OR = 2.032), T3 category (OR = 1.324), T4a category (OR = 1.945), and T4b category (OR = 2.163) (all *P* < .05) were independent hazard prognostic factors for SRC gastric cancer. OR = odds ratio, SRC = signet ring cell.

Results of Cox multivariate analysis showed that tumor size (OR = 2.032), T3 category (OR = 1.324), T4a category (OR = 1.945), and T4b category (OR = 2.163) were independent hazard prognostic factors for SRC GC (all *P* < .05). No significant difference was found between the 2 groups in terms of age, sex, and grade (all *P* < .05, Table 2, Fig. 2).

**Table 2:**
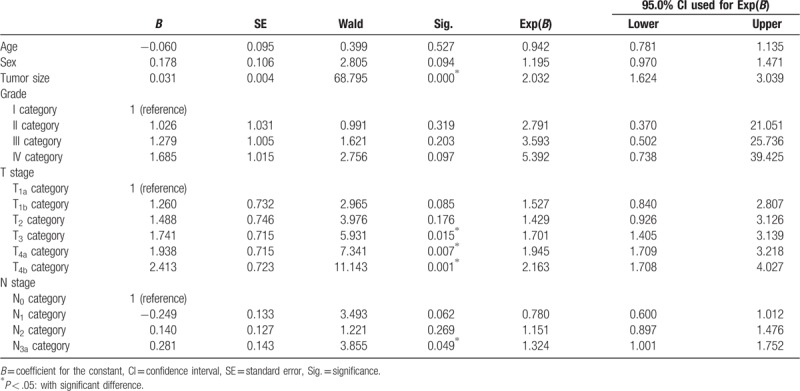
Multiple COX analysis for gastric cancer patients with D_2_ resection.

## 4 Discussion

Currently, the treatments for SRC GC remain controversial. To our knowledge, this was the first study to systematically assess the clinical significance of tumor size in the detection of SRC GC using data from the SEER database, which covers a broad geographic area. Over the last 2 decades, the incidence of SRC increased from 6% to 20%. In this study, SRC accounted for 9.17% of all GC cases, consistent with reports of previous studies from France and the United States.^[9,10]^

We evaluated the data of 946 patients with SRC GC included in the SEER database. The X-tile plot cut-off for tumor size was 49 mm. This value was used to divide patients into 2 groups comprising those with tumors larger (n = 601; large-sized tumor group) and smaller than this cut-off (n = 345; small-sized tumor group).

The tumor load was associated with tumor size. Consistent with reports of previous studies, a larger tumor size was strongly associated with a greater invasion depth, worse histological grade, peritoneal and neural metastases, more advanced Borrmann-type GC, and a higher incidence of lymph node metastases.^[11–16]^ According to the clinical baseline, patients with SRC pN(+) GC were more frequently categorized as T4 (50.74% vs 32.18%) and larger tumor sizes than patients with node-negative disease (all *P* < .05). These findings add weight to the above conclusion.

Patients with SRC GC who presented with a larger tumor size had a poorer prognosis. In this study, the 3-year OS for the large-sized tumor group (≥49 mm) was inferior to that of the small-sized tumor group (<49 mm) (44.96% vs 30.63%, *P* < .05). This finding indicates that patients with SRC GC who have larger tumors are at a higher risk of infiltrative growth, lymph node metastasis, and distant metastasis characterized by peritoneal dissemination, all of which are associated with a poor prognosis.^[17–20]^ Neoadjuvant therapy should be administered regardless of the T and N staging because SRC GC has a distinct biological behavior, presents as a large-sized tumor (≥49 mm), and is associated with worse outcomes. Therefore, the results of this study have important implications for the clinical management of SRC GC, including the type of surgical treatment, use of conversion therapy, and follow-up strategy.

In addition to prognosis, the GC tumor size is associated with LN metastasis according to several recent studies.^[16,21–24]^ For example, a Cox proportional hazards model was used to divide GC patients into 2 subgroups (tumor sizes of ≤10 vs >10 cm).^[25]^ In addition, tumor size is also associated with poorer survival. A study including 1697 patients identified tumor size as an independent prognostic factor in patients with advanced gastric cancer, as a statistically significant difference in the survival rate was observed depending on the tumor size (≤6 vs >6 cm).^[26]^ These results are similar to our findings. Moreover, a study by Thibault showed that the prognosis of early gastric SRC carcinoma was better than that of non-SRC carcinoma, whereas the prognosis of advanced stage SRC gastric carcinoma (especially T3 disease) was worse than that of non-SRC carcinoma. It is possible that infiltration is obvious in an advanced case of SRC carcinoma, and that lymph node metastasis is more likely to occur. Accordingly, patients differed markedly with respect to the likelihood of LN metastases. A multivariate analysis further verified that the tumor size (OR = 2.032) was an independent prognostic factor for SRC GC. Based on our data, greater attention should be paid to patients with GC who present with large-sized tumors, as they face a higher risk of LN metastasis.

This study had a few inherent limitations. First, this was a retrospective study and was subject to the limitations of the study design. In the future, a prospective study design will be necessary. Moreover, the study data were obtained from the SEER database between 2004 and 2011, during which period not all stage 3 and many stage 2 patients were recommended to receive neoadjuvant therapy. Clinically, it is difficult to determine lymph node metastasis preoperatively, and it is difficult to diagnose T2 and T3 SRC GC via imaging. Therefore, the TNM stages of many cases were defined via postoperative pathology. Second, the SEER database does not contain detailed information about the therapy administered to each patient. We note that advances have been made in the palliative treatment of GC. Additionally, the diverse and multi-sequential chemotherapy received by patients led to research bias. Third, this study had a small sample size. After stratification by tumor size, each subgroup contained an inadequate number of samples. This likely weakened the statistical power of our analysis. Finally, the accuracy of survival benefits may have been exaggerated because of the absence of treatment for tumor metastases and the lack of data on the burden of tumor metastasis.

## 5 Conclusions

In conclusion, our results indicate that a distinct feature of SRC GC is the presence of a large-sized tumor (≥49 mm), which is associated with a worse outcome. SRC GC patients with large-sized tumors had a 2.032 times risk of mortality. In addition, SRC patients with larger tumors are at higher risk for infiltrative growth, lymph node metastasis, and distant metastasis.

## Acknowledgments

The authors would like to thank Xuefei Cheng, Wei Zeng, and Lihang Liu for their skillful technical assistance and to Jinhua Chen and Lingfeng Wang for their assistance with statistical analysis.

## Author contributions

**Conceptualization:** Weihua Li.

**Data curation:** Shaoxin Cai, Changshun Yang.

**Formal analysis:** Shaoxin Cai, Changshun Yang.

**Software:** Shaoxin Cai.

**Writing – original draft:** Liyuan Zhou.

**Writing – review & editing:** Liyuan Zhou, Weihua Li, Shaoxin Cai, Yi Liu, Zhizun Lin.
